# The Comparison of Predicting Factors and Outcomes of MINOCA and STEMI Patients in the 5-Year Follow-Up

**DOI:** 10.3390/jpm13050856

**Published:** 2023-05-19

**Authors:** Patryk Buller, Adam Kern, Maciej Tyczyński, Wojciech Rosiak, Włodzimierz Figatowski, Robert J. Gil, Jacek Bil

**Affiliations:** 1 Department of Cardiology, Provincial Integrated Hospital, 09-400 Plock, Poland; patryk@buller.com.pl (P.B.); figel@wp.pl (W.F.); 2 Department of Cardiology and Internal Medicine, University of Warmia and Mazury, 10-082 Olsztyn, Poland; 3 Department of Invasive Cardiology, Centre of Postgraduate Medical Education, 02-508 Warsaw, Poland; 4 Department of Internal Medicine, Independent Public Complex of Healthcare Institutions, 09-300 Zuromin, Poland; 5 State Medical Institute of the Ministry of Interior and Administration, 02-508 Warsaw, Poland

**Keywords:** myocardial infarction with non-obstructive coronary arteries, acetylcholine, INOCA

## Abstract

The long-term outcomes of patients with myocardial infarction with non-obstructive coronary arteries (MINOCA) are still not well known. This study aimed to compare the characteristics and outcomes between MINOCA and STEMI patients in a 5-year follow-up. Between 2010 and 2015 we identified 3171 coronary angiography procedures performed due to acute coronary syndrome, from which 153 had a working MINOCA diagnosis, and the final diagnosis of MINOCA was ascribed to 112 (5.8%) patients. Additionally, we matched 166 patients with STEMI and obstructive coronary arteries as the reference group. In MINOCA patients (mean age of 63 years), there were more females (60% vs. 26%, *p* < 0.001), and patients presented most frequently with NSTEMI (83.9%). Patients with MINOCA had more frequent atrial fibrillation (22% vs. 5.4%, *p* < 0.001) and higher left ventricular ejection fraction (59 ± 10% vs. 54 ± 10%, *p* < 0.001) compared to STEMI patients. We observed only a trend for a higher rate of MACE in STEMI patients at 5 years (11.6% vs. 18.7%, HR 1.82, 95% CI 0.91–3.63, *p* = 0.09). In multivariable Cox regression, only beta-blocker use was a protective factor (a trend observed), with HR 0.33, 95% CI 0.10–1.15, *p* = 0.082 of future MACE. The outcomes of MINOCA and STEMI patients were comparable in the 5-year follow-up.

## 1. Introduction

Myocardial infarction with non-obstructive coronary arteries (MINOCA) is defined by clinical and laboratory evidence of myocardial infarction (MI) and no significant coronary artery narrowing (lesions with diameter stenosis < 50%). However, one must stress that this is a heterogeneous syndrome, possibly driven by several different pathophysiological pathways [1].

In recent years, we have witnessed a huge surge of interest in MINOCA, focusing on a personalized approach to diagnostics and treatment strategy in this group of patients. In various studies, the prevalence of MINOCA is reported to be within the range of 5–15% of the total population undergoing coronary angiography due to MI [2]. As stated earlier, MINOCA can be caused by various diseases that are often hard to identify, such as coronary microcirculatory dysfunction, plaque rupture/erosion, or epicardial coronary spasm [3]. In addition, the underlying inflammatory comorbidities are not indifferent to the MINOCA development [4], e.g., recent STEMI prevalence with no culprit lesions was reported in 1/5 of COVID-19 patients [5]. Importantly, MINOCA’s perception has changed significantly. Physicians, especially cardiologists, have stopped treating MINOCA as a benign disorder and recognized that those patients have a worse prognosis and similar long-term outcomes as patients with obstructive coronary artery disease. For example, in a median follow-up of nearly 20 years, an Italian study showed that recurrent MI (17.3% vs. 25.4%, *p* = 0.18), ischemic stroke (9.5% vs. 3.7%, *p* = 0.12) or all-cause death (14.1% vs. 20.7%, *p* = 0.26) were comparable between MINOCA patients and patients with obstructive coronary artery disease [6]. Several other studies also presented similar results [7,8,9,10]. However, some others showed that MINOCA patients had a better prognosis than patients with MI and obstructive coronary arteries [11,12].

The study aimed to compare the characteristics and outcomes between MINOCA and ST-elevation MI (STEMI) with obstructive coronary artery patients in a 5-year follow-up.

## 2. Materials and Methods

### 2.1. Study Design and Participants

The data were obtained retrospectively from the hospital database. First, we analyzed all patients who underwent coronary angiography due to myocardial infarction, i.e., NSTEMI or STEMI. We then identified patients with coronary angiography with non-obstructive coronary arteries (lesions < 50% of diameter stenosis) (MINOCA working diagnosis). For the final analysis, we included patients with a final diagnosis of MINOCA after excluding other potential causes such as pulmonary embolism or myocarditis. The definition of MINOCA was verified according to the diagnostic criteria enclosed in the European Society of Cardiology guidelines on NSTE-ACS [13]. Additionally, we identified a STEMI group with obstructive disease that were matched based on age and the event date.

This study compared various baseline demographic and clinical characteristics, laboratory data, and clinical outcomes at a 5-year follow-up between MINOCA and STEMI patients.

### 2.2. Data Collection

We retrieved demographic, clinical, periprocedural, and laboratory data from the hospital database. We took into consideration the following comorbidities: arterial hypertension, dyslipidemia, diabetes mellitus, peripheral artery disease, atrial fibrillation, chronic kidney disease (defined as eGFR < 60 mL/min/1.73 m^2^), prior coronary artery bypass grafting (CABG), prior PCI (percutaneous coronary intervention), prior MI, and clinical data associated with MI: MI type, disease severity, treatment strategy, and periprocedural complications. Additionally, we gathered information on echocardiographic parameters (left ventricular ejection fraction, left ventricular end-diastolic diameter, intraventricular septal diameter, left atrial diameter) and laboratory findings assessed at admission: complete blood count with differential (WBC—white blood cells, RBC—red blood cells, Hgb—hemoglobin, PLT—platelets), alanine aminotransferase (ALT), creatinine, troponin T, C-reactive protein (CRP), eGFR, glucose, glycated hemoglobin (HbA1c), lipid profile, N-terminal pro-B-type natriuretic peptide (NT-proBNP), thyroid-stimulating hormone (TSH), and uric acid. We also gathered information on medications at discharge. 

### 2.3. Study Endpoints

The primary study endpoint was to compare the 5-year rate of major cardiovascular adverse events (MACE) defined as joined rates of cardiac death, MI, and recurrent hospitalization due to angina. The secondary endpoints included all-cause death, cardiac death, MI, PCI, and recurrent hospitalization due to angina rates at 1, 2, 3, 4, and 5 years.

### 2.4. Statistical Methods

Descriptive statistics were presented: mean, standard deviation, minimum, median, interquartile range, and maximum for continuous variables; count and percent for categorical variables. Pearson’s Chi-squared test or Fisher’s exact test was performed to compare categorical variables between the two groups (e.g., MINOCA and STEMI patients). Fisher’s exact test was used when at least one of the subgroups had a count = 0. The Wilcoxon rank sum test was performed to compare continuous variables between two groups (e.g., MINOCA and STEMI patients). A *p*-value < 0.05 was considered statistically significant.

Kaplan-Meier estimators with 95% CI were calculated to compare 5-year survival curves for various endpoints between groups (e.g., MINOCA and STEMI patients).

If a given endpoint occurred for a particular patient more than once in a 5-year follow-up period, then the survival time was assumed as the time to the first occurrence of this endpoint. Notably, in the case of MACE (a composite endpoint), survival time was assumed as the time to the first occurrence of either cardiac death, myocardial infarction, or angina pectoris hospitalization.

Univariable and multivariable Cox regression (the Cox proportional hazards model) was performed to compare survival rates between groups. The multivariable Cox regression model was chosen in stepwise selection with a backward elimination algorithm with a significance level = 0.3. Results regarding the Hazard Ratio (HR) and 95% confidence intervals for HR were presented.

Statistical analyses were performed using R software version 4.2.1 (23 June 2022 ucrt)—“Funny-Looking Kid” Copyright (C) 2022 The R Foundation for Statistical Computing Platforma: x86_64-w64-mingw32/x64 (64-bit).

## 3. Results

### 3.1. Baseline Characteristics

**Figure 1:**
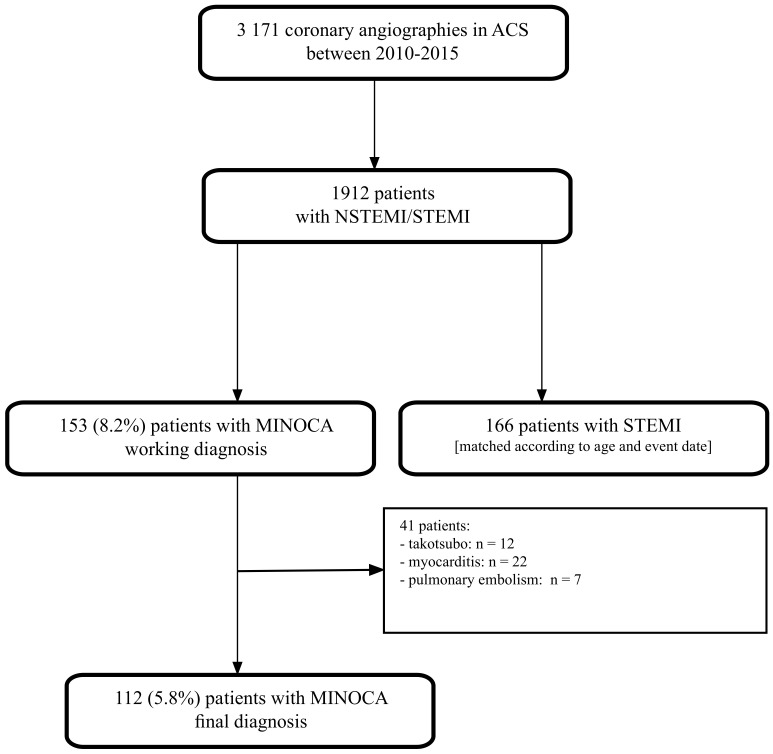
Study flowchart. ACS—acute coronary syndrome; NSTEMI—non-ST-elevation myocardial infarction; STEMI—ST-elevation myocardial infarction, MINOCA—myocardial infarction with non-obstructive coronary arteries.

Between 2010 and 2015, we identified 3171 coronary angiography procedures performed due to acute coronary syndrome, of which 153 had a working MINOCA diagnosis, and the final diagnosis of MINOCA was ascribed to 112 (5.8%) patients (Figure 1). Additionally, we matched 166 patients with STEMI and obstructive coronary arteries as the reference group.

The baseline characteristics are presented in Table 1, and angiographic data are shown in Table 2. In MINOCA patients, there were more females (60% vs. 26%, *p* < 0.001), and patients presented most frequently with NSTEMI (83.9%). Patients with MINOCA had more frequent atrial fibrillation (22% vs. 5.4%, *p* < 0.001) and higher left ventricular ejection fraction (59 ± 10% vs. 54 ± 10%, *p* < 0.001) compared to STEMI patients. In STEMI patients, we observed higher rates of dyslipidemia (25% vs. 37%, *p* = 0.031) and prior MI (0 vs. 5.4%, *p* = 0.012). Most patients with MINOCA had no coronary lesions (45.5%) or lesions < 30% in diameter (36.6%). In STEMI patients, significant lesions (>50% in diameter) were present in only one artery in 42.2% of cases (one-vessel disease) and in two arteries in 37.3% of patients (two-vessel disease).

Table 3 presents laboratory findings at admission. Interestingly, MINOCA patients had lower glucose, creatine levels, LDL, and triglycerides, whereas they had higher levels of HDL. Also, MINOCA patients had lower levels of troponin T, mainly within the range of 0–500 ng/mL (59.8%), whereas STEMI patients had troponin T levels mainly within the range of 2501–10,000 ng/mL (54.8%), *p* < 0.001.

### 3.2. Management at Discharge

All included patients were discharged. Table 4 presents medications prescribed at discharge. STEMI patients received ASA (95% vs. 100%, *p* = 0.004), clopidogrel (73% vs. 100%), beta-blocker (79% vs. 90%, *p* = 0.009), ACE inhibitor (73% vs. 93%, *p* < 0.001), nitrates (55% vs. 77%, *p* < 0.001) and statins (91% vs. 99%, *p* < 0.001) more frequently. MINOCA patients received more frequent Ca-blockers (25% vs. 9.6%, *p* < 0.001).

### 3.3. Outcomes at 5 Years

**Figure 2:**
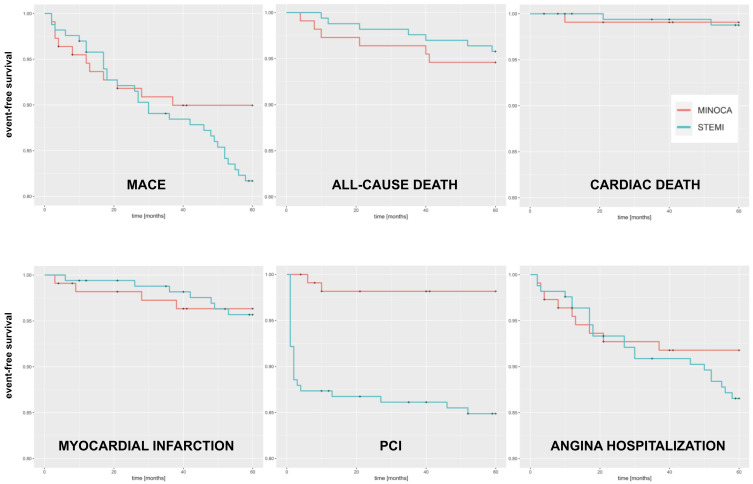
Kaplan-Meier curves for 5-year outcomes. PCI: percutaneous coronary intervention.

Survival rates at 5 years are presented in Table 5, and Kaplan-Meier curves are shown in Figure 2. We only observed a statistically significant difference for PCI, which was higher for STEMI patients (1.8% vs. 15.1%, HR 9.0, 95% CI 2.13–38.0, *p* = 0.003), and a trend for a higher rate of MACE in STEMI patients (11.6% vs. 18.7%, HR 1.82, 95% CI 0.91–3.63, *p* = 0.09). We provide the data on all-cause death, cardiac death, and myocardial infarction rates at the follow-up with a median of 9 years (Table S1 and Figure S1) in the Supplementary Material. We observed no statistically significant differences in terms of those outcomes between MINOCA and STEMI patients.

### 3.4. Predictor Factors for MACE at 5-Year Follow-Up

Additionally, we performed a Cox regression analysis to identify the predictive factors for MACE in MINOCA and STEMI patients separately. Table 6 shows the analysis for MINOCA patients. Here, only a beta blocker was a protective factor (a trend observed)—HR 0.33, 95% CI 0.10–1.15, *p* = 0.082. Table 7 shows the analysis for STEMI patients. Here, the use of an angiotensin receptor blocker (HR 9.35, 95% CI 1.03–84.6, *p* = 0.047) was the significant predictive factor, and a trend was observed for male sex HR 2.87, 95% CI 0.98–8.46, *p* = 0.056.

## 4. Discussion

We analyzed 3171 myocardial infarction cases and identified 112 patients with a final diagnosis of MINOCA. The mean MINOCA incidence was 5.8%. We observed similar 5-year outcomes between the MINOCA and STEMI patients. No statistically significant differences were observed in MACE rates.

The MINOCA incidence (5.8%) in our paper was similar to values provided in other reports, i.e., between 5% and 15% [10,14,15,16]. In addition, the recent paper on MINOCA incidence in Poland just before the COVID-19 pandemic (6.3%) and during the COVID-19 pandemic (5.9%) showed similar results [17]. The rate of MINOCA in the MINOCA-TR Registry (10.3%) was slightly higher, but similarly to ours, women dominated in the MINOCA population (our study—60%, MINOCA-TR—89%) [18].

In our study, MINOCA causes were rarely thoroughly investigated. This also reflected the approach to those patients a couple of years ago (2010–2015), when not much attention was provided to patients with no lesions in coronary arteries unless there was a suspicion of an underlying disease, e.g., myocarditis or a plaque rupture (suggestive coronary angiography view) [19]. Bucciarelli et al. recently showed that cardiac magnetic resonance (cMR) could really change the diagnosis [20]. They observed that 1/3 of ischemic MINOCA cases were initially treated as myocarditis, but after including cMR results, different management was adopted in 22% of subjects. Unfortunately, too few patients are still referred to cMR. This may be associated with costs issue as well as with cMR availability. Not every hospital has this imaging modality. Also, it was recently shown that other techniques could be helpful in the diagnosis of patients with ischemia and non-obstructive coronary arteries. We refer to intracoronary ECG [21] or comprehensive microcirculatory and epicardial vessel bed assessment as suggested by the recent European Society of Cardiology guidelines [13,22,23,24].

In our paper, we observed similar outcomes between MINOCA and STEMI at 5 years. One must also stress that the results were quite favorable, with low rates of all-cause death (5.4% vs. 4.2%), cardiac death (0.9% vs. 1.2%), recurrent myocardial infarction (3.6% vs. 4.2%) or MACE (11.6% vs. 18.7%). The rates of repeated hospitalizations due to angina in MINOCA and STEMI patients were 8.1% vs. 13.3%, respectively. The relative low incidence of comorbidities could have influenced this finding, with only 42% of patients having arterial hypertension, or 37% of patients having dyslipidemia in the STEMI group. However, as shown in the Supplementary Materials, the outcomes at the very long follow-up (median: MINOCA—9 years, STEMI—9.8 years) were much worse (all-cause death rate—17.9% vs. 24.1%, *p* = 0.61; cardiac death rate—9.8% vs. 16.9%, *p* = 0.31 and MI rate—14.3% vs. 21.1%, *p* = 0.41). Nevertheless, once again, no statistical difference was observed between the MINOCA and STEMI patients.

Interestingly, in the previously published study on MINOCA in Poland, no statistical difference in 12-month all-cause mortality in patients with MINOCA before and during the COVID-19 pandemic was observed, but the values were higher than in our study (total population 9.9%; 9.2% (2019) vs. 11.0% (2020), *p* = 0.09) [17]. Quesada et al. showed that at five years, the all-cause death rate was higher in ST-elevation MINOCA cases than in STEMI patients (18% vs. 15%, *p* = 0.033). In the propensity score-matched cohort, ST elevation MINOCA patients demonstrated a 1.4-fold (95%CI 1.04–1.89, *p* = 0.028) higher risk of 5-year all-cause death than STEMI patients [25]. However, this study gathered patients from 2003, and many advancements have been observed in pharmacotherapy and invasive treatment strategies since then which could have influenced the long-term outcomes. Recently, Zalewska-Adamiec et al. presented interesting results on MINOCA patients’ survival in a 3-year follow-up. The all-cause death rate of MINOCA patients was 16.85%. However, they differentiated two subgroups with eGFR < 60 and ≥ 60 mL/min/1.73 m^2^. At 12 months, the all-cause death was 26.42% vs. 7.20% (*p* = 0.0004), and after 36 months, it was 33.96% vs. 9.6% (*p* < 0.0001) [26]. In our study, only 3.6% of patients had chronic kidney disease. This can also be one of the factors explaining our favorable outcomes.

In our study, we reported that in MINOCA patients, only beta-blockers could potentially decrease the risk of MACE at 5 years (a trend observed: HR 0.33, 95% CI 0.10—1.15, *p* = 0.082). However, in STEMI patients, the potential predictor factors of MACE at 5 years were angiotensin receptor blocker use (HR 9.35, 95% CI 1.03—84.6, *p* = 0.047) and male sex (a trend: HR 2.87, 95% CI 0.98—8.46, *p* = 0.056). As mentioned earlier, Bucciarelli et al. showed that dyslipidemia, reduced left ventricular ejection fraction, ST-elevation at the hospitalization, and the late-gadolinium enhancement transmural pattern were the independent predictors of cardiac-related rehospitalizations in MINOCA patients [20]. In addition, complete blood count-derived indices could predict the diagnosis and outcomes, such as platelet distribution width [1]. Notably, Gabaldon-Perez et al. observed that elderly patients with MINOCA had a better prognosis than those with obstructive coronary arteries; however, this only applied to those with no lesions in the coronary arteries [11].

Chen et al. showed no differences in outcomes between non-ST-elevation or ST-elevation groups during a 4-year follow-up [27]. This was also true in our study. Chen et al. also identified multivariable predictors of MACE. In the non-ST-elevation group, there were Killip grades ≥ 2, reduced beta-blocker use during hospitalization, and higher levels of low-density lipoprotein cholesterol. The reduced beta-blocker use was the sole independent factor in the ST-elevation group [27]. We also observed that using beta-blockers in MINOCA patients had a beneficial effect in terms of MACE. Nevertheless, Li et al. recently showed that ST-elevation MINOCA patients demonstrated lower risks for MACE at a 2-year follow-up [7]. Here, we should also mention the paper by Jedrychowska et al., in which the authors designed a risk score for predicting MINOCA amid overall STEMI patients [28]. And, interestingly, Stepien et al. showed that the MINOCA treatment and outcomes were not affected whether patients were treated during the weekend (free days) or working days [10].

### Study Limitations

This study has several limitations. First, this was a retrospective study; therefore, residual confounding factors may exist. Second, we could not systematically establish real MINOCA causes, since cardiac magnetic resonance or IVUS/OCT was rarely used. And, finally, we included all MINOCA patients we could identify; therefore, no sample size calculation was performed; however, relatively small populations might have caused no statistically significant evident differences in the outcomes between MINOCA and STEMI patients. Nevertheless, most papers on MINOCA present the results on 100–200 patient populations.

## 5. Conclusions

We observed the frequency of MINOCA patients at 5.8%. In addition, we observed comparable long-term outcomes between MINOCA and STEMI patients. No statistically significant differences were observed in MACE rates (11.6% vs. 18.7%, HR 1.82, 95% CI 0.91–3.63, *p* = 0.09).

## Figures and Tables

**Table 1:** Baseline characteristics.

Parameter	MINOCA *N* = 112	STEMI *N* = 166	*p*-Value
Females	67 (60%)	43 (26%)	<0.001
Age [years]	63 ± 14	61 ± 11	0.2
Body mass index [kg/m^2^]	27.9 ± 5.8	28.1 ± 4.8	0.3
Myocardial infarction type at presentation			
NSTEMI	94 (83.9%)	-	-
STEMI	18 (16.1%)	166 (100)	<0.001
Arterial hypertension	59 (53%)	69 (42%)	0.068
Diabetes type 2	15 (13%)	29 (17%)	0.4
Dyslipidemia	28 (25%)	62 (37%)	0.031
Prior myocardial infarction	0 (0%)	9 (5.4%)	0.012
Prior PCI	0 (0%)	3 (1.8%)	0.3
Prior CABG	0 (0%)	1 (0.6%)	>0.9
Chronic kidney disease	4 (3.6%)	9 (5.4%)	0.5
Atrial fibrillation	25 (22%)	9 (5.4%)	<0.001
Peripheral artery disease	1 (0.9%)	5 (3.0%)	0.4
Smoking	13 (12%)	27 (16%)	0.3
Echocardiographic parameters			
LVEDd [mm]	48.7 ± 6.0	51.4 ± 6.4	<0.0001
IVSd [mm]	11.1 ± 2.6	11.3 ± 2.0	0.056
LA [mm]	39.8 ± 6.4	3.96 ± 5.6	>0.9
LVEF [%]	59 ± 10	54 ± 10	<0.001

NSTEMI—non-ST-elevation myocardial infarction, STEMI—ST-elevation myocardial infarction, PCI—percutaneous coronary intervention, CABG—coronary artery bypass grafting, LVEDd—left ventricular end-diastolic diameter, IVSd—interventricular septal diameter; LA—left atrial diameter; LVEF—left ventricular ejection fraction.

**Table 2:** Angiographic data.

Parameter	MINOCA *N* = 112	STEMI *N* = 166
Coronary lesions		
No lesions	51 (45.5%)	-
<30%	41 (36.6%)	-
30–50%	20 (17.9%)	-
Intravascular imaging use	5 (4.5%)	20 (12.1%)
Infarct-related artery		
LAD	-	79 (47.6%)
LCx	-	19 (11.4%)
RCA	-	68 (41.0%)
TIMI before PCI		
0	-	142 (85.5%)
1	-	20 (12.1%)
2	-	4 (2.4%)
3	-	0
TIMI post PCI		
0	-	1 (0.6%)
1	-	1 (0.6%)
2	-	4 (2.4%)
3	-	160 (96.4%)
Disease advancement		
1VD	-	70 (42.2%)
2VD	-	62 (37.3%)
3VD/LM	-	34 (20.5%)
Stent implanted		
BMS	-	117 (70.5%)
DES	-	45 (27.1%)
No stent	-	4 (2.4%)

LAD—left anterior descending artery; LCx—left circumflex artery; RCA—right coronary artery; PCI—percutaneous coronary intervention; VD—vessel disease; BMS—bare metal stent; DES—drug-eluting stent.

**Table 3:** Laboratory findings at admission.

Parameter	MINOCA N = 112	STEMI N = 166	*p*-Value
White blood cells [10^9^/L]	9.8 ± 4.1	11.7 ± 3.8	<0.001
Hemoglobin [g/dL]	13.84 ± 1.47	14.55 ± 1.40	<0.001
Red blood cells [10^12^/L]	4.55 ± 0.48	4.76 ± 0.47	0.002
Platelets [10^9^/L]	250 ± 80	261 ± 72	0.13
Glucose [mmol/L]	7.43 ± 2.48	9.08 ± 3.56	<0.001
HbA1c [%]	5.91 ± 0.37	7.99 ± 2.28	0.053
NT-proBNP [pg/mL]	3747 ± 5149	5622 ± 5323	0.005
C-reactive protein	3.5 ± 9.5	5.6 ± 7.1	0.3
AST [U/L]	74 ± 128	143 ± 144	0.092
ALT [U/L]	41 ± 28	54 ± 30	0.13
Total cholesterol [mmol/L]	4.78 ± 1.11	5.24 ± 1.13	0.004
HDL [mmol/L]	1.58 ± 0.65	1.23 ± 0.35	<0.001
LDL [mmol/L]	2.59 ± 1.04	3.22 ± 1.00	<0.001
Triglycerides [mmol/L]	1.37 ± 0.60	1.72 ± 0.98	0.002
Creatine [µmol/l]	86 ± 32	91 ± 19	<0.001
TSH [µU/mL]	1.57 ± 1.42	1.50 ± 1.61	0.4
Uric acid [µmol/L]	406 ± 111	414 ± 98	0.6
Maximal troponin T [ng/mL]			
0–500	67 (59.8%)	16 (9.6%)	<0.001
501–2500	39 (34.8%)	45 (27.2%)	
2501–10,000	6 (5.4%)	91 (54.8%)	
10,000+	0	14 (8.4%)	

ALT—alanine aminotransferase, AST—asparagine aminotransferase, HbA1c—glycated hemoglobin, NT-proBNP—N-terminal pro-B-type natriuretic peptide, TSH—thyroid-stimulating hormone, Troponin T reference values: 0—0.037 ng/mL, and the cut-off for MI: 0.1 ng/mL.

**Table 4:** Medications at discharge.

Parameter	MINOCA *N* = 112	STEMI *N* = 166	*p*-Value
ASA	105 (95%)	166 (100%)	0.004
Clopidogrel	81 (73%)	166 (100%)	<0.001
Beta-blocker	88 (79%)	150 (90%)	0.009
Ca-blocker	28 (25%)	16 (9.6%)	<0.001
ACE inhibitor	81 (73%)	155 (93%)	<0.001
Angiotensin receptor blocker	5 (4.5%)	2 (1.2%)	0.12
Diuretic	25 (23%)	19 (11%)	0.013
Trimetazidine	2 (1.8%)	4 (2.4%)	>0.9
Nitrates	61 (55%)	127 (77%)	<0.001
Vitamin K antagonist	13 (12%)	4 (2.4%)	0.002
Novel oral anticoagulant	4 (3.6%)	0 (0%)	0.025
Statin	101 (91%)	165 (99%)	<0.001

ASA—aspirin, ACE—angiotensin-converting enzyme.

**Table 5:** 5-year outcomes in MINOCA vs. STEMI.

Parameter	MINOCA *N* = 112	STEMI *N* = 166	HR	95% CI	*p*
All-cause death	6 (5.4%)	7 (4.2%)	0.77	0.26–2.29	0.6
Cardiac death	1 (0.9%)	2 (1.2%)	1.31	0.12–14.5	0.8
Myocardial infarction	4 (3.6%)	7 (4.2%)	1.15	0.34–3.92	0.8
Percutaneous intervention	2 (1.8%)	25 (15.1%)	9.0	2.13–38.0	0.003
Hospitalization due to angina	9 (8.1%)	22 (13.3%)	1.62	0.75–3.53	0.2
MACE	13 (11.6%)	31 (18.7%)	1.82	0.91–3.63	0.09

MACE—major adverse cardiovascular event.

**Table 6:** Univariable and multivariable Cox regression for MINOCA patients.

	Univariable Analysis			Multivariable Analysis		
Characteristic	HR	95% CI	*p*	HR	95% CI	*p*
Sex						
Female	—	—				
Male	0.55	0.15–2.07	0.375			
Age	1.01	0.97–1.05	0.762			
Body mass index	0.95	0.84–1.08	0.429			
Coronary arteries lesions						
No lesions	—	—				
<30%	0.78	0.19–3.27	0.735			
30–50%	1.27	0.25–6.53	0.778			
Final diagnosis						
STEMI	—	—				
NSTEMI	0.87	0.19–4.04	0.862			
Arterial hypertension	2.52	0.67–9.49	0.173	2.90	0.76–11.0	0.118
Dyslipidemia	0.64	0.14–2.94	0.562			
Diabetes	2.38	0.63–8.98	0.201			
Smoking	0.70	0.09–5.50	0.737			
Atrial fibrillation	1.35	0.36–5.09	0.658			
NT-proBNP	1.00	1.00–1.00	0.397			
LDL	0.68	0.38–1.22	0.194			
Creatine	1.00	0.99–1.02	0.664			
Left ventricular ejection fraction	0.97	0.92–1.02	0.239	0.97	0.92–1.02	0.221
Clopidogrel	0.57	0.17–1.96	0.374			
Beta-blocker	0.43	0.12–1.46	0.173	0.33	0.10–1.15	0.082
Ca blocker	1.78	0.52–6.07	0.359			
ACE inhibitor	1.00	0.27–3.78	0.997			
Angiotensin receptor blocker	2.21	0.28–17.3	0.450			
Statin	0.95	0.12–7.44	0.963			

NSTEMI—non-ST-elevation myocardial infarction, STEMI—ST-elevation myocardial infarction, N-terminal pro-B-type natriuretic peptide, ACE—angiotensin-converting enzyme.

**Table 7:** Univariable and multivariable Cox regression for STEMI patients.

	Univariable Analysis			Multivariable Analysis		
Characteristic	HR	95% CI	*p*	HR	95% CI	*p*
Sex						
Female	-	-				
Male	2.38	0.83–6.82	0.106	2.87	0.98–8.46	0.056
Age	0.99	0.96–1.03	0.633			
Body mass index	0.98	0.90–1.06	0.602			
IRA location						
LAD	-	-		-	-	-
LCx	2.19	0.77–6.21	0.142	2.20	0.74–6.58	0.156
RCA	1.29	0.59–2.82	0.529	1.35	0.58–3.16	0.490
Disease advancement						
1VD	-	-				
2VD	1.51	0.66–3.43	0.331			
3VD/LM	1.53	0.58–4.03	0.385			
Stent type						
BMS	-	-				
DES	0.60	0.25–1.47	0.264	0.51	0.19–1.35	0.174
Arterial hypertension	0.81	0.39–1.71	0.583			
Dyslipidemia	1.31	0.64–2.69	0.467			
Diabetes	0.98	0.38–2.57	0.973			
Smoking	0.76	0.26–2.17	0.606			
Chronic kidney disease	1.50	0.36–6.32	0.577			
Peripheral artery disease	1.30	0.18–9.55	0.797			
Prior myocardial infarction	0.67	0.09–4.94	0.697			
NTproBNP	1.00	1.00–1.00	0.852			
LDL	1.29	0.89–1.88	0.181	1.22	0.85–1.77	0.281
Creatine	1.00	0.98–1.02	0.803			
Left ventricular ejection fraction	1.02	0.98–1.06	0.431			
Beta-blocker	3.28	0.45–24.1	0.243	3.78	0.50–28.4	0.197
Ca blocker	0.66	0.16–2.77	0.569			
ACE inhibitor	2.10	0.29–15.4	0.466			
Angiotensin receptor blocker	4.88	0.66–35.9	0.119	9.35	1.03–84.6	0.047

IRA: infarct-related artery; LAD: left anterior descending artery; LCx: left circumflex artery; RCA: right coronary artery; VD: vessel disease; LM: left main; BMS: bare metal stent; DES: drug-eluting stent; N-terminal pro-B-type natriuretic peptide, ACE: angiotensin-converting enzyme.

## Data Availability

Raw data are available from the corresponding author upon request.

## Supplementary Materials

The following supporting information can be downloaded at: https://www.mdpi.com/article/10.3390/jpm13050856/s1, Figure S1: Kaplan-Meier curves on long-term outcomes (follow-up of median 9 years); Table S1: 9-year follow-up data.
