# Supplementary material for: The Comparison of Predicting Factors and Outcomes of MINOCA and STEMI Patients in the 5-Year Follow-Up
Source: J Pers Med. 2023 May 19;13(5):856. doi: 10.3390/jpm13050856 (PMC10223109; doi:10.3390/jpm13050856)
Supplement: Supplementary file 1 [file jpm-13-00856-s001.zip › jpm-2351252-supplementary.pdf]

Supplementary material

As supplementary material we present data for longer follow-up up to 31.12.2022 assessing all-cause death, cardiac death, and myocardial infarction rates (Table S1). Additionally, Figure S1 presents Kaplan-Meier curves for those endpoints.
Although numerically higher rates for assessed endpoints were observed in STEMI patients, no statistically significant differences were confirmed (Figure S1).

Table S1. 9-year follow-up data

	**MINOCA**<br>**N = 112**	**STEMI**<br>**N = 166**
Lost to follow-up*	8 (7.1%)	22 (13.3%)
Median follow-up (IQR) [months]	109.5 (96.8 – 129)	117 (97-139)
min. – max. follow-up [months]	4 - 153	10 - 153
All-cause death rate	20 (17.9%)	40 (24.1%)
Cardiac death rate	11 (9.8%)	28 (16.9%)
Myocardial infarction rate	16 (14.3%)	35 (21.1%)

*for first 60 months no lost to follow-up cases were reported. Lost to follow-up was observed for repeated longer observation > 60 months.

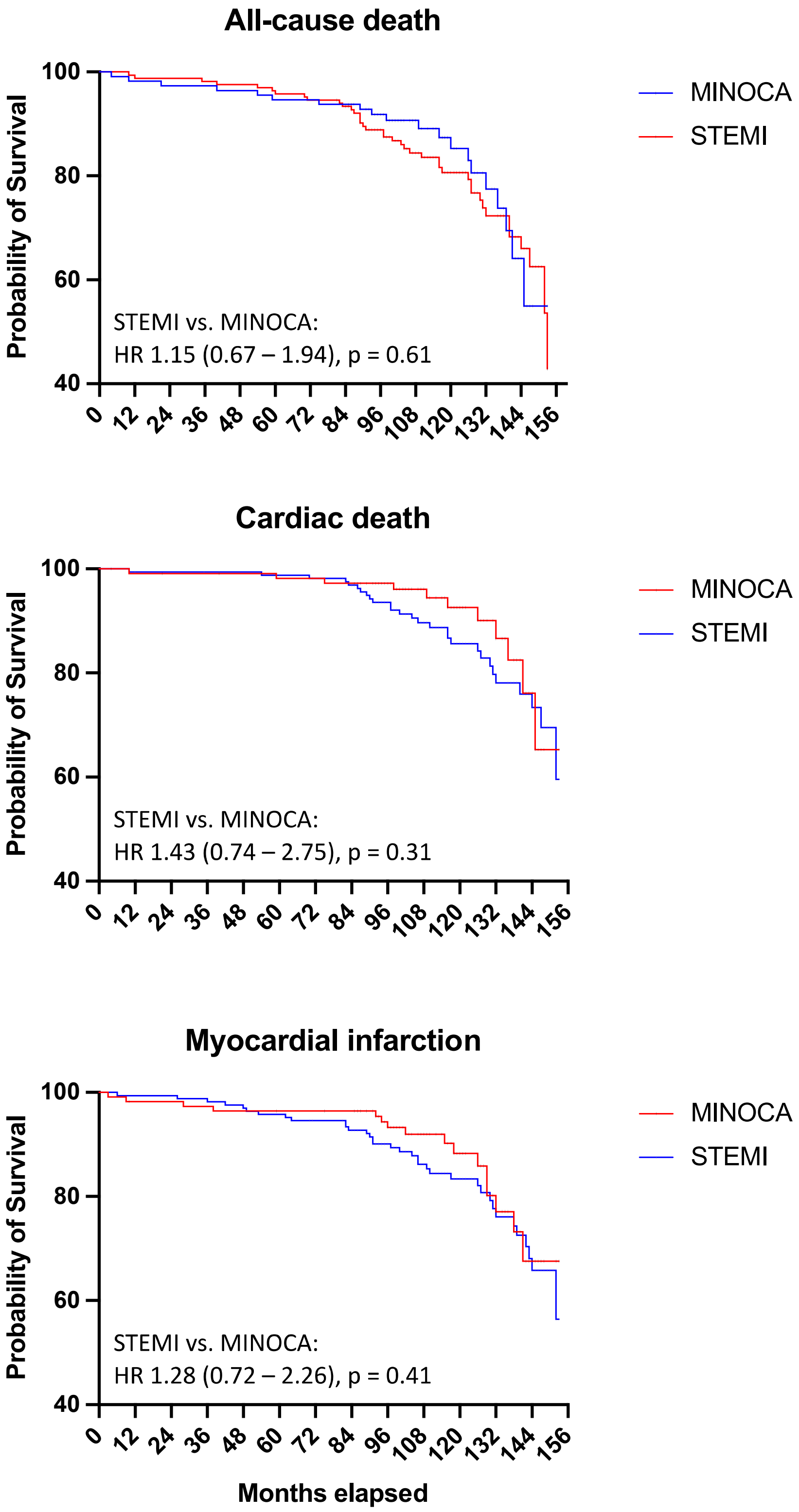

Numbers at risk

Months	0	12	24	36	48	60	72	84	96	108	120	132	144
MINOCA	112	110	109	109	108	106	98	97	95	85	59	41	27
STEMI	166	165	165	164	162	159	135	133	126	120	101	71	46

Figure S1. Kaplan-Meier curves on long-term outcomes (follow-up of median 9 years)
